# Epidemiological Classification Changes and Incidence of Early-Onset Colorectal Cancer

**DOI:** 10.1001/jamanetworkopen.2025.41732

**Published:** 2025-11-05

**Authors:** Valérie Jooste, Jean-Baptiste Nousbaum, Arnaud Alves, Anne-Sophie Woronoff, Guy Launoy, Florence Molinié, Emmanuel Desandes, Patricia Delafosse, Brigitte Tretarre, Emilie Mercier, Côme Lepage, François Ghiringhelli, Anne-Marie Bouvier

**Affiliations:** 1 Digestive Cancer Registry of Burgundy, Dijon, France; 2 Centre Hospitalier Universitaire (CHU) Dijon Bourgogne, Dijon, France; 3 Institut National de la Santé et de la Recherche Médicale (INSERM) Research Center LNC-UMR1231, Dijon, France; 4 Université Bourgogne Europe, Dijon, France; 5 FRANCIM Network, Toulouse, France; 6 Finistère Digestive Cancer Registry, EA 7479 SPURBO, University Hospital, Brest, France; 7 ANTICIPE U1086 INSERM-UCN, Centre François Baclesse, Normandie Unicaen, Caen, France; 8 Calvados Digestive Cancer Registry, University Hospital Centre, Caen, France; 9 Doubs Cancer Registry, Besançon University Hospital, Besançon, France; 10 Loire-Atlantique and Vendée Cancer Registry, Nantes University Hospital, Nantes, France; 11 French National Registry of Childhood Solid Tumors, CHU Nancy, Nancy, France; 12 Centre de Recherche en Epidémiologie et en Statistique Sorbonne-Paris Cité (CRESS), Unité Mixte de Recherche 1153, INSERM, Université Paris-Descartes, Paris, France; 13 Isère Cancer Registry, Grenoble-Alpes University Hospital, Grenoble, France; 14 Hérault Cancer Registry, Montpellier, France; 15 Cancer Biology Transfer Platform, Department of Tumor Biology and Pathology, Georges-François Leclerc Anticancer Center, Unicancer, Dijon, France; 16 Department of Medical Oncology, Centre Georges-François Leclerc, Dijon, France; 17 Department of Gastroenterology, Dijon University Hospital, Dijon, France

## Abstract

**Importance:**

Previous studies showed an increasing incidence of early-onset colorectal cancer (EOCRC) during a period when cancer registration guidelines were changed. None of these studies simultaneously compared the patterns in colorectal adenocarcinoma (ADC) and neuroendocrine neoplasms (NENs) according to age and stage extension.

**Objective:**

To describe patterns in colorectal cancer incidence according to histopathological type and tumor extension at diagnosis in individuals younger than 50 years compared with older patients.

**Design, Setting, and Participants:**

This cohort study used data from the French Network of Cancer Registries (FRANCIM) on inhabitants of 7 French administrative areas. The cohort included individuals newly diagnosed with invasive colorectal cancer from 2004 to 2021. This period covered the change in NEN classification published in 2013. Statistical analysis was conducted from November 2024 to March 2025.

**Main Outcomes and Measures:**

Age-specific incidence rates of ADC and NENs were primary outcomes. Incidence was calculated and modeled to estimate annual percent changes (APCs) using a flexible multivariable Poisson model. In a sensitivity analysis, Joinpoint regressions consolidated the results.

**Results:**

A total of 63 780 patients (35 266 males [55.3%]; mean [SD] age, 63.8 [12.8] years) were diagnosed with all colorectal cancer histologic types (CRC-All). The incidence of EOCRC increased in females (APC, 2.9%; 95% CI, 1.6%-4.3%) and males (APC, 2.6%; 95% CI, 1.2%-4.0%) aged 15 to 39 years but remained stable in those aged 40 to 49 years. In both sexes and all ages, the rates of ADC did not vary while NEN rates increased. In EOCRC, the increase in NENs was marked from 2004 to 2013 (between 10.1% [95% CI, 4.0%-16.5%] per year and 12.6% [95% CI, 7.2%-18.2%] per year) and disappeared thereafter (ranging from −1.1% [95% CI, −1.9% to −0.2%] per year to −1.7% [95% CI, −3.0% to −0.4%] per year). It affected the incidence of colorectal cancer only for patients aged 15 to 39 years, for whom the proportion of NENs was high (29.7% [278 of 935]) compared with 5.7% (132 of 2333) of patients in the 40 to 49 years age class and 1.4% (856 of 60 512) of patients in the 50 years or older age class). In EOCRC, ADC M1 (distant metastasis) incidence increased in both sexes over the entire study period (between 1.2% [95% CI, 0.3%-2.1%] per year and 2.3% [95% CI, 1.0%-3.6%] per year between 2013 and 2021).

**Conclusions and Relevance:**

In this cohort study, the 2013 change in NENs classification had a prominent role in EOCRC incidence patterns. Public health decision-makers should consider this artifactual increase.

## Key Points

**Question:**

How does changing cancer registration guidelines affect the estimation of colorectal cancer incidence?

**Findings:**

In this cohort study of 63 780 patients with colorectal cancer, changes in the definition of neuroendocrine neoplasms (NENs) affected the incidence of colorectal cancer only for people aged 15 to 39 years, for whom the proportion of NENs was sufficient to change incidence. Time-series patterns for nonmetastatic NENs were similar across all age classes, while metastatic NENs remained rare.

**Meaning:**

Findings of this study suggest that public health decision-makers should consider the artifactual increase in NEN incidence amid increasing colorectal cancer rates, especially regarding the current debate about initiating colorectal cancer screening at an earlier age.

## Teaser

This cohort study examines age-specific incidence rates of colorectal adenocarcinoma and neuroendocrine neoplasms in individuals with invasive colorectal cancer.

## Introduction

Colorectal cancer is a major global health challenge, currently ranked as the third most commonly diagnosed cancer worldwide.^1^ In France, the estimated number of new cases diagnosed in 2023 was 48 000. In high-income countries, the incidence and mortality of colorectal cancer often reflect a decrease or stabilization in people older than 50 years, partly as a result of the progressive implementation of cancer mass screening programs. Although the majority of cases are associated with older age classes, there is growing concern globally over the greater incidence of early-onset colorectal cancer (EOCRC), defined as diagnosis in individuals younger than 50 years. Using population-based data from the International Agency for Research on Cancer Incidence in Five Continents, Sung et al^2^ recently described a rising EOCRC incidence between 2008 and 2017 in 27 of 50 countries and territories examined, either exclusive to early-onset disease or faster than the increase in older adults in 20 of the 27 countries. Although some molecular sequence variations have been exhibited in younger adults compared with those in older adults, underlying biomolecular profiles and pathological features remain incompletely understood.^3^ EOCRC mostly presents with advanced disease and unfavorable histopathological features.

However, reliable population-based data on the epidemiological characteristics of EOCRC remain scarce,^4^ especially studies simultaneously considering the histopathological type and tumor stage in their description of EOCRC incidence patterns. On the proposal from the European Neuroendocrine Tumour Society, the World Health Organization made some definition changes and indicated the tools of grading and staging.^5^ This recommendation was published in the fourth edition of the World Health Organization blue book *Classification of Tumors of the Digestive System* (viewed as the reference for registration tools in cancer registries) in 2010.^6^ From then on, carcinoid tumors not otherwise specified—designated with *International Classification of Diseases for Oncology, Third Edition (ICD-O-3)* code 8240 as benign or borderline—were reportable as invasive tumors for all sites except the appendix, an exception removed in 2013.^7,8^

Comprehensive population-based studies are essential to provide unbiased pictures of time patterns in incidence according to clinical characteristics of EOCRC. The purpose of this study was to describe time patterns in colorectal cancer incidence according to histopathological type and tumor extension at diagnosis in individuals younger than 50 years compared with older patients, over an 18-year period in a well-defined French population.

## Methods

### Data Sources and Study Design

This cohort study was based on data from cancer registries in the French Network of Cancer Registries (FRANCIM). A representative of each participating registry was involved in the study and approved the use of FRANCIM data. The Comité Consultatif sur le Traitement de l’Information en Matière de Recherche dans le Domaine de la Santé provided ethics approval for this study, and the Commission Nationale Informatique et Libertés provided legal framework and data protection for each cancer registry. Patient informed consent was not obtained because French authorities do not require supplementary authorization for studies based on cancer registries. We followed the Strengthening the Reporting of Observational Studies in Epidemiology (STROBE) reporting guideline.

Colorectal cancer cases are informed by many sources: public and private pathology laboratories, regional health system databases, and public and private hospital discharge databases. French registries do not record incident cases that are informed only by death certificates. Death certificates mentioning bowel cancer and colorectal cancer cases that were not registered during life were individually traced back. The small number of untraceable cases were not registered.

All incident solid colorectal cancers diagnosed between 2004 and 2021 in inhabitants of 7 French administrative areas (Calvados, Côte-d’Or, Doubs, Finistère, Hérault, Isère, and Saône-et-Loire) were included in this cohort study. Rules of the International Agency for Research on Cancer allow for the inclusion of only 1 cancer per body site during a patient’s lifetime, unless there are multiple cancers of different histological types. If there were multiple synchronous colorectal tumors in the same patient, the most advanced stage was included.

According to *ICD-O-3*, the cancer sites were the colon (*ICD-O-3* code C18), including the appendix (*ICD-O-3* code C18.1), rectosigmoid junction (*ICD-O-3* code C19), and rectal ampulla (*ICD-O-3* code C20).^8^ Adenocarcinoma (ADC) codes were 8140-8149, 8160-8163, 8190-8221, 8250-8384, 8390-8420, 8430, 8440-8490, 8500-8543, 8550-8552, and 8570-8576. Neuroendocrine neoplasms (NENs) included all neuroendocrine tumors, carcinomas, and mixed types; NEN codes were as follows: 8013, 8150-8158, and 8240-8249. Other rare histological types were included only when considering all colorectal cancer histological types (CRC-All). Stage at diagnosis for ADC was coded using the pathological *TNM Classification of Malignant Tumours*^9^: stages I (T1-2, N0M0), II (T3-4, N0M0), III (all T, N1-2M0), and IV (M1). Additionally, unresected, nonmetastatic colorectal ADC was classified as the M0 unresected stage. For some analyses, stages I, II, III, and M0 unresected were grouped into ADC M0. For NENs, stage at diagnosis was defined as NEN M0 for no distant metastasis and NEN M1 for distant metastasis. Metastatic status was unknown for 1285 ADCs and 77 NENs; therefore, they were excluded from stage-specific analyses. Age at diagnosis was classified into 15 to 39 years, 40 to 49 years, and 50 years or older age classes. For the descriptive analysis of specific incidence rates, the 18-year time frame between 2004 and 2021 was divided into 6 periods of 3 years.

### Statistical Analysis

Incidence rates were calculated for each of the 3 age classes as crude rates per 100 000 person-years. Incidence was modeled by age class for the whole group (CRC-All), for each histopathological outcomes, and according to tumor extension. All analyses were performed by sex and for pooled males and females. Year of diagnosis was considered in the flexible multivariable Poisson model as a continuous variable with a nonlinear association with incidence, without a priori knowledge of cutoff year. Annual percent changes (APCs) were calculated by age class from 2004 to 2021, 2004 to 2013, and 2013 to 2021 using model-based estimates of age-specific incidence rates for the years 2004, 2013, and 2021. Their 95% CIs were estimated using the delta method.^10^

As a sensitivity analysis, APCs were also estimated using Joinpoint analyses, which considered the outcome of year of diagnosis as linear by segment. Sensitivity analyses were performed for males and females pooled for CRC-All and for each histopathological and stage group.

To focus on the cohort effect, an age-period-cohort model was fitted for CRC-All and for each histopathological outcomes, including the drift term in the cohort effect. This parametrization allowed postestimation of the cohort effect relative to the reference cohort (1980), of the incidence rate by age given for the 1980 reference cohort and adjusted for the period effect, and of the period effect constrained to have 0 slope and to be 0 on average (on the log scale).^11^

Statistical computations were performed using Stata 18 (StataCorp LLC)^12^ and the Joinpoint Regression Program, version 5.3.0.0 (National Cancer Institute).^13^ These analyses were conducted from November 2024 to March 2025.

## Results

Of the 63 780 patients with CRC-All (28 514 females [44.7%], 35 266 males [55.3%]; mean [SD] age, 63.8 [12.8] years), 3268 (5.1%) were diagnosed before age 50 years and were considered to have EOCRC. Of these patients, 935 (28.6%) were ages 15 to 39 years and 2333 (71.4%) were ages 40 to 49 years. The proportion of NENs among patients with CRC-All was 29.7% (278 of 935) in the age class 15 to 39 years, 5.7% (132 of 2333) in the age class 40 to 49 years, and 1.4% (856 of 60 512) in the age class 50 years or older. The corresponding proportions for ADC were 69.5% (650 of 695), 93.6% (2183 of 2333), and 98.3% (59 485 of 60 512). Cancer was located in the appendix in 267 of 935 patients (28.6%) aged 15 to 39 years, 95 of 2333 patients (4.1%) aged 40 to 49 years, and 507 of 60 512 patients (0.8%) aged 50 years or older. In the age class 15 to 39 years, 93 of 420 patients (22.1%) had cancer located in the appendix before 2013, and the number increased to 174 of 515 patients (33.8%) after 2013. In the age class 40 to 49 years, these proportions were 3.1% (35 of 1121 patients) and 4.9% (60 of 1212 patients), respectively. In the appendix, NENs were represented by 240 of 267 patients (89.9%) in the age class 15 to 39 years, 59 of 95 patients (62.1%) in the age class 40 to 49 years, and 159 of 507 patients (31.4%) in the age class 50 years or older.

Table 1 provides a description of the 63 584 patients with colorectal ADC and NENs. For ADC, the male to female ratio was 1.0 in the 15- to 39-years age class, 1.1 in the 40- to 49-years age class, and 1.3 in the 50 years or older age class; for NENs, the ratios were 0.6, 1.2, and 1.1, respectively. Age distribution varied according to histopathological type: for the NEN group, 278 of 1266 patients (22.0%) were aged 15 to 39 years and 132 of 1266 patients (10.4%) were aged 40 to 49 years, while the ADC group consisted of 650 of 62 318 patients (1.0%) aged 15 to 39 years and 2183 of 62 318 patients (3.5%) aged 40 to 49 years.

**Table 1.** Description of Colorectal Adenocarcinoma and Neuroendocrine Neoplasms Diagnosed Between 2004 and 2021 by Age Class and Histopathological Type

Characteristic	Patients, No. (%)					
	ADC			NEN		
	Aged 15-39 y	Aged 40-49 y	Aged ≥50 y	Aged 15-39 y	Aged 40-49 y	Aged ≥50 y
All	650 (1.0)	2183 (3.5)	59 485 (95.5)	278 (22.0)	132 (10.4)	856 (67.6)
Sex						
Male	322 (0.9)	1139 (3.3)	33 098 (95.8)	99 (15.9)	71 (11.4)	451 (72.6)
Female	328 (1.2)	1044 (3.8)	26 387 (95.1)	179 (27.8)	61 (9.5)	405 (62.8)
Period						
2004-2006	97 (1.0)	353 (3.7)	9112 (95.3)	25 (23.6)	14 (13.2)	67 (63.2)
2007-2009	97 (1.0)	355 (3.6)	9448 (95.4)	16 (15.8)	10 (9.9)	75 (74.3)
2010-2012	128 (1.3)	363 (3.6)	9723 (95.2)	53 (26.0)	19 (9.3)	132 (64.7)
2013-2015	107 (1.0)	384 (3.7)	9985 (95.3)	60 (22.0)	31 (11.4)	182 (66.7)
2016-2018	118 (1.0)	369 (3.2)	10 914 (95.7)	54 (17.5)	31 (10.1)	223 (72.4)
2019-2021	103 (1.0)	359 (3.3)	10 303 (95.7)	70 (25.5)	27 (9.9)	177 (64.6)
Extension at diagnosis^a^						
M0	425 (0.9)	1513 (3.4)	42 893 (95.7)	240 (27.2)	101 (11.5)	540 (61.3)
TNM stage I	117 (0.9)	453 (3.5)	12 204 (95.5)	NA	NA	NA
TNM stage II	140 (0.9)	459 (2.9)	15 035 (96.2)	NA	NA	NA
TNM stage III	159 (1.2)	572 (4.3)	12 493 (94.5)	NA	NA	NA
M0 unresected	9 (0.3)	29 (0.9)	3161 (98.8)	NA	NA	NA
M1	214 (1.3)	653 (4.0)	15 335 (94.6)	9 (2.9)	20 (6.5)	279 (90.6)

Abbreviations: ADC, adenocarcinoma; M0, no distant metastasis; M1, distant metastasis; NA, not applicable; NEN, neuroendocrine neoplasm.

^a^ Extension at diagnosis unknown in 2.1% (1285 in ADC; 77 in NEN).

Among the patients presenting with metastasis at diagnosis, 214 of 16 202 (1.3%) ADCs and 9 of 308 (2.9%) NENs occurred in the 15- to 39-years age class. In patients with ADC, the proportions of M1 were 32.9% (214 of 650), 29.9% (653 of 2183), and 25.8% (15 335 of 59 485) in the age classes 15 to 39 years, 40 to 49 years, and 50 years or older. In patients with NENs, these proportions were 3.2% (9 of 278), 15.2% (20 of 132), and 32.6% (279 of 856), respectively.

### Observed Patterns of Colorectal Cancer Incidence

**Figure 1.**
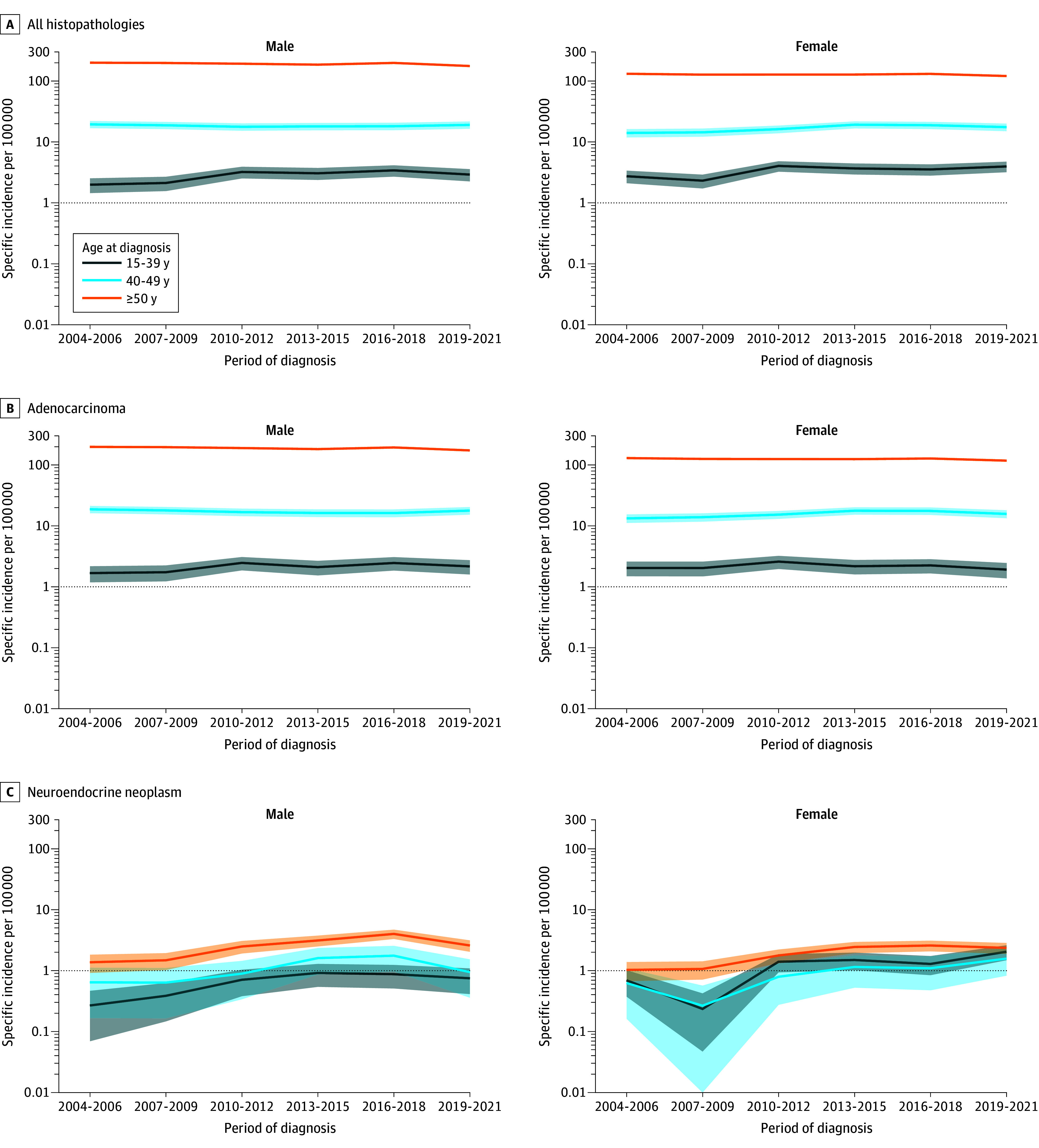
Observed Specific Incidence of Colorectal Cancer by Age Class, Sex, and Histopathological Type

Observed age-specific incidence patterns of CRC-All by sex from 2004 to 2021 are presented in Figure 1A and eTable 1 in Supplement 1. The incidence of CRC-All slightly increased for males and females aged 15 to 39 years until the 2010 to 2012 period and then stabilized, whereas the incidence was stable throughout the study for those aged 40 to 49 years. For males and females older than 50 years, the incidence of CRC-All was stable in the first four 3-year periods and then tended to decrease.

Time-series patterns in observed incidence by age differed greatly according to histopathological type (Figure 1B and 1C; eTable 1 in Supplement 1). Over the first 5 periods, ADC incidence varied little in both sexes, except for a slight increase in the 15- to 39-years age class. The incidence of ADC decreased during the 2019 to 2021 period in all age classes. In contrast, NENs incidence increased until the 2013 to 2015 period in all age classes, especially 15 to 39 years.

### Model-Based Patterns of Colorectal Cancer Incidence 

**Figure 2.**
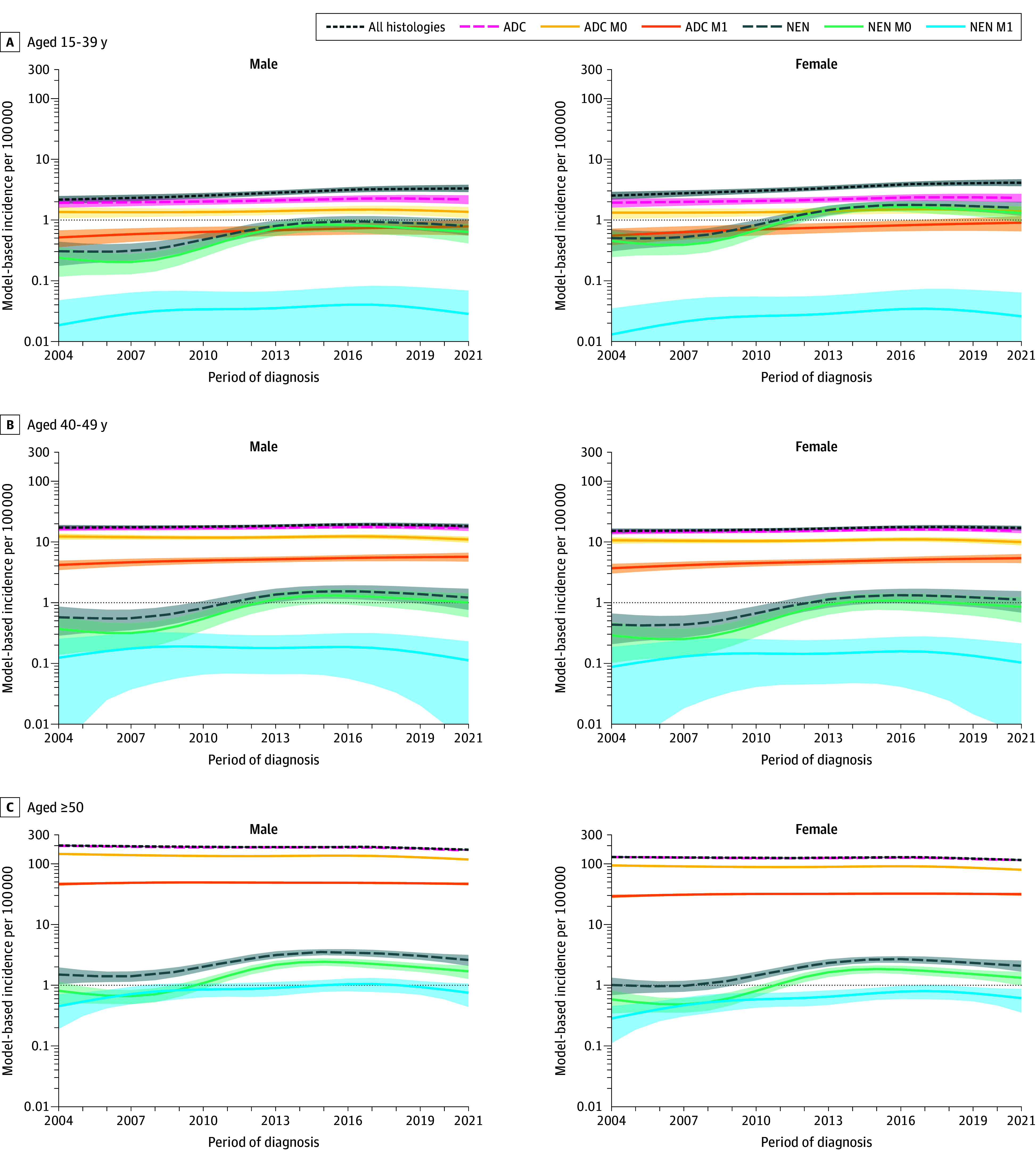
Model-Based Specific Incidence of Colorectal Cancer by Histopathological Type, Tumor Extension, Sex, and Age Class ADC indicates adenocarcinoma; NEN, neuroendocrine neoplasm; M0, no distant metastasis; and M1, distant metastasis.

Time-series patterns in model-based incidence by sex and age varied according to histopathological type and tumor extension (Figure 2; eTables 2 to 4 in Supplement 1). Figure 2 shows, in male and female with CRC-All, an increase in early onset, particularly in those aged 15-39 years, and a decrease in those 50 years or older. The incidence of ADC did not vary in EOCRC and decreased over the entire study period in those aged 50 years or older. In both the 15- to 39-years and 40- to 49-years age classes, the incidence of ADC M0 was stable while the incidence of M1 increased slightly and regularly between 2004 and 2021. In all age classes, the incidence of NEN M0 was stable between 2004 and 2007, increased sharply between 2007 and 2013, and then stabilized and slightly decreased thereafter.

To quantify these variations in incidence, mean changes were calculated for the entire study period and for mid-periods (2004-2013 and 2013-2021) (Table 2 and Table 3). In CRC-All, from 2004 to 2021, APC was higher in females (2.9%; 95% CI, 1.6%-4.3%) and males (2.6%; 95% CI, 1.2%-4.0%) aged 15 to 39 years than in 40- to 49-year-old females (0.6; 95% CI, −0.3% to 1.5%) and males (0.3%; 95% CI, −0.6% to 1.2%). The increase in incidence was more marked during the 2004-2013 period than the 2013-2021 period. Inversely, the incidence of CRC-All decreased in patients 50 years or older over the 2004 to 2013 period, mostly in males (APC, −0.7%; 95% CI,−1.1% to −0.2%), and during the 2013 to 2021 period in females (APC, −1.1%; 95% CI,−1.5% to −0.7% ) and males (APC, −1.4%; 95% CI, −1.8% to −1.0%). The increase in NENs was marked from 2004 to 2013 (between 10.1% [95% CI, 4.0%-16.5%] and 12.6% [95% CI, 7.2%-18.2%] per year). The incidence decreased thereafter, ranging from −1.1% (95% CI, −1.9% to −0.2%) to −1.7% (95% CI, −3.0% to −0.4%) per year. In patients with ADC, TNM stage IV incidence increased over the entire study period in EOCRC, mostly in females aged 15 to 39 years (APC, 2.9%; 95% CI, 0.1%-5.9%) and aged 40 to 49 years (APC, 2.3%; 95% CI, 0.6%-4.0%). Between 2013 and 2021, the APC ranged in both sexes between 1.2% (95% CI, 0.3%-2.1%) and 2.3% (95% CI, 1.0%-3.6%). The incidence of ADC in TNM stages I, II, and III did not vary over time in EOCRC (Table 2 and Table 3).

**Table 2.** Model-Based Annual Percent Changes in Colorectal Cancers by Histopathological Type and Tumor Extension by Age Class and Sex

Histopathological type and tumor extension by age class	APC (95% CI), %					
	Males			Females		
	2004-2013	2013-2021	2004-2021	2004-2013	2013-2021	2004-2021
**CRC-All**						
15-39 y	3.0 (1.5 to 4.5)	2.2 (0.9 to 3.5)	2.6 (1.2 to 4.0)	3.3 (1.8 to 4.8)	2.5 (1.2 to 3.9)	2.9 (1.6 to 4.3)
40-49 y	0.7 (−0.3 to 1.6)	−0.1 (−1.0 to 0.8)	0.3 (−0.6 to 1.2)	1.0 (−0.0 to 2.0)	0.2 (0.6 to 1.1)	0.6 (−0.3 to 1.5)
≥50 y	−0.7 (−1.1 to −0.2)	−1.4 (−1.8 to −1.0)	−1.0 (−1.3 to −0.7)	−0.3 (−0.8 to 0.1)	−1.1 (−1.5 to −0.7)	−0.7 (−1.0 to −0.4)
**ADC**						
15-39 y	1.1 (−0.7 to 2.8)	0.5 (−0.8 to 1.8)	0.8 (−0.8 to 2.4)	1.3 (−0.4 to 3.1)	0.8 (−0.5 to 2.1)	1.1 (−0.5 to 2.7)
40-49 y	0.3 (−0.7 to 1.3)	−0.3 (−1.2 to 0.6)	−0.0 (−0.9 to 0.9)	0.6 (−0.4 to 1.6)	−0.0 (−0.9 to 0.9)	0.3 (−0.6 to 1.2)
≥50 y	−0.8 (−1.2 to −0.3)	−1.3 (−1.7 to −0.9)	−1.0 (−1.3 to −0.7)	−0.5 (−0.9 to 0.0)	−1.0 (−1.4 to −0.6)	−0.7 (−1.1 to −0.4)
**ADC M0**						
15-39 y	0.4 (−1.7 to 2.5)	−0.4 (−1.7 to 0.9)	0.0 (−1.9 to 2.0)	0.7 (−1.4 to 2.8)	−0.1 (−1.4 to 1.2)	0.3 (−1.6 to 2.3)
40-49 y	−0.3 (−1.5 to 0.9)	−1.1 (−1.9 to −0.2)	−0.7 (−1.7 to 0.4)	−0.0 (−1.2 to 1.2)	−0.8 (−1.7 to 0.1)	−0.4 (−1.5 to 0.7)
≥50 y	−0.9 (−1.4 to −0.3)	−1.6 (−2.0 to −1.3)	−1.2 (−1.6 to −0.9)	−0.6 (−1.1 to −0.0)	−1.4 (−1.8 to −1.0)	−1.0 (−1.3 to −0.6)
**ADC M1**						
15-39 y	3.1 (0.0 to 6.2)	1.8 (0.5 to 3.2)	2.5 (−0.4 to 5.4)	3.5 (0.4 to 6.7)	2.3 (1.0 to 3.6)	2.9 (0.1 to 5.9)
40-49 y	2.4 (0.5 to 4.4)	1.2 (0.3 to 2.1)	1.8 (0.1 to 3.6)	2.9 (0.9 to 4.8)	1.6 (0.7 to 2.5)	2.3 (0.6 to 4.0)
≥50 y	0.6 (−0.3 to 1.5)	−0.6 (−1.0 to −0.2)	0.0 (−0.6 to 0.6)	1.0 (0.1 to 2.0)	−0.2 (−0.6 to 0.2)	0.5 (−0.2 to 1.1)
**NEN**						
15-39 y	11.4 (5.9 to 17.1)	−0.1 (−1.4 to 1.2)	5.8 (2.0 to 9.8)	12.6 (7.2 to 18.2)	1.0 (−0.3 to 2.3)	7.0 (3.3 to 10.7)
40-49 y	10.1 (4.0 to 16.5)	−1.2 (−2.1 to −0.4)	4.6 (−0.0 to 9.4)	11.2 (5.0 to 17.9)	−0.2 (−1.0 to 0.7)	5.7 (1.0 to 10.7)
≥50 y	8.7 (4.2 to 13.5)	−2.4 (−2.8 to −2.0)	3.3 (0.5 to 6.2)	9.9 (5.2 to 14.8)	−1.4 (−1.8 to −1.0)	4.5 (1.6 to 7.4)
**NEN M0**						
15-39 y	13.2 (6.4 to 20.4)	−1.7 (−3.0 to −0.4)	5.9 (1.3 to 10.7)	13.7 (7.2 to 20.6)	−1.2 (−2.5 to 0.1)	6.4 (2.2 to 10.8)
40-49 y	13.3 (5.6 to 21.7)	−1.5 (−2.4 to −0.7)	6.1 (0.4 to 12.1)	13.9 (6.0 to 22.4)	−1.1 (−1.9 to −0.2)	6.6 (0.8 to 12.7)
≥50 y	11.6 (5.6 to 17.9)	−3.1 (−3.4 to −2.7)	4.4 (0.8 to 8.2)	12.1 (6.0 to 18.6)	−2.6 (−3.0 to −2.2)	4.9 (1.2 to 8.8)
**NEN M1**						
15-39 y	7.4 (−9.1 to 27.0)	−2.7 (−3.9 to −1.4)	2.5 (−11.7 to 19.0)	9.1 (−7.9 to 29.2)	−1.2 (−2.4 to 0.1)	4.1 (−10.4 to 21.0)
40-49 y	4.2 (−8.0 to 18.0)	−5.6 (−6.5 to −4.8)	−0.6 (−10.5 to 10.5)	5.8 (−6.7 to 20.0)	−4.2 (−5.0 to −3.3)	1.0 (−9.2 to 12.3)
≥50 y	8.0 (−0.6 to 17.3)	−2.2 (−2.6 to −1.8)	3.0 (−2.2 to 8.6)	9.7 (0.7 to 19.4)	−0.7 (−1.1 to −0.3)	4.7 (−0.9 to 10.5)

Abbreviations: ADC, adenocarcinoma; APC, annual percent change; CRC-All, all colorectal cancer histologic types; M0, no distant metastasis; M1, distant metastasis; NEN, neuroendocrine neoplasm.

**Table 3.** Model-Based Annual Percent Changes in Colorectal Adenocarcinoma by TNM Stage by Age Class and Sex

TNM stage by age class	APC (95% CI) , %					
	Males			Females		
	2004-2013	2013-2021	2004-2021	2004-2013	2013-2021	2004-2021
**Stage I**						
15-39 y	0.5 (−3.5 to 4.6)	−0.2 (−3.7 to 3.5)	0.2 (−3.5 to 4.0)	1.6 (−2.4 to 5.8)	1.0 (−2.6 to 4.6)	1.3 (−2.4 to 5.2)
40-49 y	−1.5 (−3.6 to 0.7)	−2.1 (−4.0 to −0.1)	−1.8 (−3.7 to 0.2)	−0.4 (−2.5 to 1.9)	−1.0 (−2.9 to 1.0)	−0.7 (−2.6 to 1.3)
≥50 y	−0.9 (−1.9 to 0.1)	−1.6 (−2.4 to −0.7)	−1.2 (−1.9 to −0.6)	0.2 (−0.9 to 1.3)	−0.5 (−1.4 to 0.5)	−0.1 (−0.9 to 0.6)
**Stage II**						
15-39 y	0.2 (−3.4 to 3.9)	−1.2 (−4.4 to 2.2)	−0.5 (−3.8 to 3.0)	0.3 (−3.3 to 4.0)	−1.0 (−4.2 to 2.3)	−0.3 (−3.7 to 3.2)
40-49 y	−0.2 (−2.3 to 2.0)	−1.5 (−3.4 to 0.4)	−0.8 (−2.7 to 1.2)	−0.1 (−2.2 to 2.1)	−1.4 (−3.3 to 0.6)	−0.7 (−2.6 to 1.3)
≥50 y	−1.0 (−1.9 to −0.1)	−2.3 (−3.1 to −1.5)	−1.6 (−2.2 to −1.0)	−0.9 (−1.8 to 0.0)	−2.2 (−3.0 to −1.4)	−1.5 (−2.1 to −0.9)
**Stage III**						
15-39 y	0.3 (−3.1 to 3.8)	−0.2 (−3.2 to 3.0)	0.1 (−3.1 to 3.3)	0.2 (−3.2 to 3.7)	−0.3 (−3.3 to 2.9)	−0.0 (−3.2 to 3.2)
40-49 y	0.2 (−1.7 to 2.2)	−0.2 (−2.0 to 1.6)	0.0 (−1.8 to 1.8)	0.1 (−1.8 to 2.1)	−0.3 (−2.1 to 1.5)	−0.1 (−1.9 to 1.7)
≥50 y	−1.4 (−2.4 to −0.5)	−1.9 (−2.8 to −1.0)	−1.7 (−2.3 to −1.0)	−1.5 (−2.5 to −0.6)	−2.0 (−2.9 to −1.1)	−1.8 (−2.4 to −1.1)
**Stage IV (M1)**						
15-39 y	3.1 (−0.0 to 6.2)	1.8 (0.5 to 3.2)	2.5 (−0.4 to 5.4)	3.5 (0.4 to 6.7)	2.3 (1.0 to 3.6)	2.9 (0.1 to 5.9)
40-49 y	2.4 (0.5 to 4.4)	1.2 (0.3 to 2.1)	1.8 (0.1 to 3.6)	2.9 (0.9 to 4.8)	1.6 (0.7 to 2.5)	2.3 (0.6 to 4.0)
≥50 y	0.6 (−0.3 to 1.5)	−0.6 (−1.0 to −0.2)	0.0 (−0.6 to 0.6)	1.0 (0.1 to 2.0)	−0.2 (−0.6 to 0.2)	0.5 (−0.2 to 1.1)

Abbreviations: APC, annual percent change; M1, distant metastasis.

As illustrated in Figure 2, the patterns of variation in NEN M0 were similar in all age classes: the APCs ranged from 11.6% (95% CI, 5.6%-17.9%) to 13.9% (95% CI, 6.0%-22.4%) between 2004 and 2013 and from −1.1% (95% CI, −1.9% to −0.2%) to −3.1% (95% CI, −3.4% to −2.7%) between 2013 and 2021 (Table 2). The incidence of NEN M1 did not vary over time. Patterns of NEN incidence were similar to patterns of NEN M0 incidence.

### Sensitivity Analysis

Pooled APCs for males and females, estimated using the flexible model, were compared with those obtained from Joinpoint analyses. The results for CRC-All and for each histopathological type and tumor extension are presented in eTable 5 in Supplement 1. The results from both analyses were similar and confirm the increased EOCRC incidence for ADC M1. The slope change in APCs found by the flexible model for NEN M0 was confirmed by the Joinpoint regression, which presented several points estimating a positive APC from 2007 to 2013 and no further increase thereafter.

### Age-Period-Cohort Model

The age-period-cohort model, including the drift term in the cohort effect, made it possible to focus on the cohort effect (eFigure in Supplement 1). It showed a robust cohort effect from the 1970 birth cohort for CRC-All. This association was robust and consistent with the 1950 cohort for NEN M0 but was not significant for ADC M0 and ADC M1. The model also confirmed that the age effect was more marked for ADC than for NENs.

## Discussion

To our knowledge, this study was the first European population–based study to compare time patterns in EOCRC incidence with those in older people, considering both histopathological type and stage at diagnosis. Our results showed an increase in the overall incidence of colorectal cancer in patients with EOCRC (aged 15-39 years at diagnosis), stabilization in the 40- to 49-years age class, and a decrease in the 50 years or older age class. The incidence of ADC M0 did not increase in each age class while that of M1 slightly increased only in males and females aged 15 to 39 years and 40 to 49 years and more markedly until 2013 than thereafter. Patterns of incidence variation over time for NENs and NEN M0 were similar in the 3 age classes, exhibiting an increase from 2007 to 2013 followed by a regular decrease thereafter. The major increase in NEN M0 incidence had an association with CRC-All incidence only for age 15 to 39 years because of the NEN burden in this age class: NENs represented 29.7% of all colorectal cancers in the 15- to 39-year age class compared with 5.7% in the 40- to 49-year age class and 1.4% in the 50 years or older age class.

Previous studies have demonstrated an increase in the incidence of colorectal cancer in those younger than 50 years in Europe, albeit without distinguishing between histological types.^14,15,16,17^ Moreover, they have used different cutoff levels for what was deemed to be early onset. Population-based literature comparing time patterns in colorectal cancer incidence between age classes, reflecting histopathological outcomes and stages at diagnosis, is still limited. Our results, which account for the proportion of ADC and NENs among colorectal cancers, are consistent with findings from earlier publications in which NENs accounted for 4% to 20% incidence before the age of 50 years.^18,19,20^ There is a wide variety of published incidence rates for NENs, presumably due to underreporting, inconsistent nomenclature, and changing classifications. These changes enlarged the registration with tumors previously considered nonmalignant. We cannot rule out that the delay in disseminating recommendations across the world varies between cancer registries, which may explain some of the geographical differences reported. We can assume that new guidelines partly explain the increase in NEN incidence seen between 2007 and 2013 in all age classes. A recent article based on SEER (Surveillance, Epidemiology, and End Results) Program data also highlighted the role of changing classification of NENs, leading to an artifactual increase in the incidence of colorectal cancer in children and young adults.^4^ For patients older than 50 years (the age of inclusion in the colorectal mass screening program in France since 2009), we cannot exclude the contribution of incidental detections of colorectal NENs through the screening program. The incidence of NENs decreased steadily after 2013, in EOCRC and in older patients. This pattern was similar in the US population^18^ and was not found in the Canadian series, which was analyzed only until 2017.^20^ Our study was unable to measure the role of risk factors such as metabolic syndrome in the incidence of NENs, which is still controversial.^21^ Further studies are needed to provide an explanation for this decrease and to examine whether the pattern is continuing.

In EOCRC, we found that while the incidence of ADC M0 was stable, metastatic M1 increased throughout the study period, less markedly at the end than at the beginning. In the 50 years or older age class, on the other hand, the incidence of metastatic and nonmetastatic ADC slightly decreased, probably due to the national screening program. Previous population-based studies have shown similar high percentages of ADC M1 in EOCRC.^22,23,24,25^ One hypothesis is that there is no screening program in young patients, that affected individuals consult later, or that practitioners do not immediately evoke colorectal cancer and attribute symptoms to irritable bowel syndrome. This hypothesis is consistent with the high proportion of colorectal ADC found by the European GEOCODE (Global Early-Onset Colorectal Cancer Database) consortium.^26^ Its European study also questioned the implications of overweight (characterized by a diet less rich in fruits and vegetables in favor of ultraprocessed foods combined with a sedentary lifestyle) for EOCRC.

Few population-based studies have analyzed temporal patterns in EOCRC according to disease extension. In Germany, Waldmann et al^17^ described increases in the incidence of early-stage (TNM stage I or II) colon and rectal cancers and of late-stage (TNM stage III or IV) rectal cancer. In the Netherlands, stage III and IV cancers substantially increased before age 40 years, while stages I and II tended to decrease over time.^15^ The authors suggested a stage migration, with enhanced diagnostic methods for locoregional and distant metastases and improved pathological techniques making it more likely for small metastases to be identified.

### Strengths and Limitations

Our analyses relied on population-based data, which are the gold standard for studying incidence patterns. We used data from the 7 well-established French cancer registries in the FRANCIM Network that routinely actively collect data on colorectal cancer stage at diagnosis, present a high level of completeness, and represent a catchment area of more than 5 million individuals. Therefore, we were able to provide an accurate overview of EOCRC by stage without using any imputation to estimate the stage.

This study included 7 administrative areas. Despite its population-based setting, its limited geographical coverage warrants caution in extrapolating results to broader or more diverse populations. Our analyses lacked the power to assess colonic or rectal cancer sublocations, and we had no information on the pretherapeutic clinical stage for rectal cancer. Due to its observational retrospective nature, this study lacked variables to assess potential risk factors for ADC and NENs (such as tumor molecular profiles, metabolic syndrome, obesity, and lifestyle or diet) and to describe symptoms, particularly those associated with ADC M1.

## Conclusions

In this population-based, retrospective cohort study describing patterns in colorectal cancer incidence between 2004 and 2021 in France, the overall incidence increased in the age class 15 to 39 years, was stable in the age class 40 to 49 years, and decreased in the age class 50 years or older. The incidence of ADC M0 did not increase in any age class, whereas ADC M1 incidence slightly increased in EOCRC for whatever sex and age class over the entire study period. The major increase in NEN M0 incidence had implications for CRC-All incidence, but only for the age class 15 to 39 years. These results contribute to recent discussions on advancing the age at which colorectal cancer screening should begin. It is important for public health decision-makers to consider the part of artifactual increase in the incidence of colorectal cancer observed in young adults over the past decades in several countries.

## Notes

Supplement 1. **eTable 1.** Observed Specific Incidence Rates (95% Confidence Interval) of Colorectal Cancer (/100,000) by Period and Histopathological Type by Sex and Age Class **eTable 2.** Model-Based Estimates of Specific Incidence Rates (95% Confidence Interval) of Colorectal Cancer (/100,000) by Histopathological Type and Tumor Extension for the Years 2004, 2013, and 2021 by Sex **eTable 3.** Model-Based Estimates of Specific Incidence Rates (95% Confidence Interval) of Colorectal Adenocarcinoma (/100,000) by TNM Stage and Age Class for the Years 2004, 2013 and 2021 by Sex **eTable 4.** Model-Based Annual Percent Changes (95% Confidence Interval) in Colorectal Adenocarcinoma by TNM Stage for the Years 2004, 2013, and 2021 by Age Class and Sex **eTable 5. **Annual Percent Change (95% Confidence Interval) in Colorectal Cancer by Age Class, Histopathological Type and Tumor Extension (Males and Females Pooled). Model-Based Estimates From Flexible Model and Joinpoint Regression **eFigure.** Effects of Age, Period and Cohort, by Histopathological Type and Tumor Extension (Age 15**-**49)

Supplement 2.
Nonauthor Collaborators

Supplement 3.
Data Sharing Statement
