# Supplementary material for: Epidemiological Classification Changes and Incidence of Early-Onset Colorectal Cancer
Source: JAMA Netw Open. 2025 Nov 5;8(11):e2541732. doi: 10.1001/jamanetworkopen.2025.41732 (PMC12590295; doi:10.1001/jamanetworkopen.2025.41732)
Supplement: Supplement 1. — eTable 1. Observed Specific Incidence Rates (95% Confidence Interval) of Colorectal Cancer (/100,000) by Period and Histopathological Type by Sex and Age Class eTable 2. Model-Based Estimates of Specific Incidence Rates (95% Confidence Interval) of Colorectal Cancer (/100,000) by Histopathological Type and Tumor Extension for the Years 2004, 2013, and 2021 by Sex eTable 3. Model-Based Estimates of Specific Incidence Rates (95% Confidence Interval) of Colorectal Adenocarcinoma (/100,000) by TNM Stage and Age Class for the Years 2004, 2013 and 2021 by Sex eTable 4. Model-Based Annual Percent Changes (95% Confidence Interval) in Colorectal Adenocarcinoma by TNM Stage for the Years 2004, 2013, and 2021 by Age Class and Sex eTable 5. Annual Percent Change (95% Confidence Interval) in Colorectal Cancer by Age Class, Histopathological Type and Tumor Extension (Males and Females Pooled). Model-Based Estimates From Flexible Model and Joinpoint Regression eFigure. Effects of Age, Period and Cohort, by Histopathological Type and Tumor Extension (Age 15-49) [file jamanetwopen-e2541732-s001.pdf]

Supplemental Online Content

Jooste V, Nousbaum JB, Alves A, et al; for the French Network of Cancer Registries (FRANCIM). Epidemiological classification changes and incidence increases in early-onset colorectal cancer. *JAMA Netw Open*. 2025;8(11):e2541732. doi:10.1001/jamanetworkopen.2025.41732

eTable 1. Observed Specific Incidence Rates (95% Confidence Interval) of Colorectal Cancer (/100,000) by Period and Histopathological Type by Sex and Age Class

eTable 2. Model-Based Estimates of Specific Incidence Rates (95% Confidence Interval) of Colorectal Cancer (/100,000) by Histopathological Type and Tumor Extension for the Years 2004, 2013, and 2021 by Sex

eTable 3. Model-Based Estimates of Specific Incidence Rates (95% Confidence Interval) of Colorectal Adenocarcinoma (/100,000) by TNM Stage and Age Class for the Years 2004, 2013 and 2021 by Sex

eTable 4. Model-Based Annual Percent Changes (95% Confidence Interval) in Colorectal Adenocarcinoma by TNM Stage for the Years 2004, 2013, and 2021 by Age Class and Sex

eTable 5. Annual Percent Change (95% Confidence Interval) in Colorectal Cancer by Age Class, Histopathological Type and Tumor Extension (Males and Females Pooled). Model-Based Estimates From Flexible Model and Joinpoint Regression

eFigure. Effects of Age, Period and Cohort, by Histopathological Type and Tumor Extension (Age 15-49)

This supplemental material has been provided by the authors to give readers additional information about their work.

© 2025 Jooste V et al. *JAMA Network Open*

eTable 1: Observed specific incidence rates (95% confidence interval) of colorectal cancer (/100,000) by period and histopathological type by sex and age class

Histology / extension	Specific incidence (95% CI) Males			Specific incidence (95% CI) Females		
	15–39 yrs	40–49 yrs	≥50 yrs	15–39 yrs	40–49 yrs	≥50 yrs
**CRC-All**						
2004–06	1.99 (1.45-2.53)	19.48 (16.86-22.10)	200.14 (194.65-205.63)	2.74 (2.10-3.38)	14.04 (11.84-16.23)	131.45 (127.43-135.48)
2007–09	2.13 (1.56-2.69)	18.79 (16.23-21.35)	198.19 (192.89-203.50)	2.31 (1.72-2.90)	14.38 (12.17-16.60)	127.26 (123.40-131.12)
2010–12	3.22 (2.53-3.92)	17.74 (15.28-20.21)	192.60 (187.51-197.69)	4.04 (3.25-4.82)	16.21 (13.87-18.55)	127.57 (123.80-131.35)
2013–15	3.06 (2.38-3.75)	18.02 (15.54-20.51)	185.94 (181.07-190.81)	3.68 (2.92-4.43)	19.19 (16.64-21.74)	127.72 (124.03-131.41)
2016–18	3.42 (2.70-4.14)	18.13 (15.59-20.67)	198.57 (193.65-203.49)	3.54 (2.80-4.28)	18.81 (16.24-21.38)	130.91 (127.25-134.57)
2019–21	2.92 (2.25-3.58)	19.09 (16.46-21.72)	176.71 (172.16-181.25)	3.96 (3.18-4.74)	17.51 (15.01-20.00)	120.78 (117.33-124.23)
**ADC**						
2004–06	1.68 (1.19-2.18)	18.74 (16.17-21.31)	198.34 (192.87-203.80)	2.04 (1.49-2.59)	13.32 (11.18-15.46)	129.97 (125.97-133.98)
2007–09	1.74 (1.23-2.25)	17.98 (15.47-20.48)	196.45 (191.17-201.74)	2.04 (1.48-2.59)	13.94 (11.76-16.12)	125.89 (122.05-129.73)
2010–12	2.48 (1.86-3.09)	16.85 (14.45-19.25)	189.72 (184.67-194.78)	2.60 (1.97-3.23)	15.33 (13.05-17.61)	125.22 (121.48-128.96)
2013–15	2.11 (1.54-2.68)	16.33 (13.96-18.69)	182.42 (177.59-187.24)	2.18 (1.60-2.76)	17.77 (15.32-20.23)	124.76 (121.11-128.40)
2016–18	2.46 (1.85-3.08)	16.28 (13.88-18.69)	194.09 (189.23-198.96)	2.25 (1.66-2.84)	17.62 (15.14-20.11)	127.90 (124.29-131.52)
2019–21	2.17 (1.60-2.74)	17.86 (15.32-20.41)	173.60 (169.10-178.11)	1.92 (1.38-2.47)	15.75 (13.38-18.11)	117.94 (114.53-121.34)
**NEN**						
2004–06	0.27 (0.07-0.47)	0.64 (0.17-1.12)	1.37 (0.92-1.83)	0.69 (0.37-1.01)	0.63 (0.16-1.09)	1.03 (0.67-1.38)
2007–09	0.39 (0.15-0.63)	0.64 (0.16-1.11)	1.48 (1.02-1.94)	0.24 (0.08-0.42)	0.27 (0.01-0.57)	1.07 (0.71-1.42)
2010–12	0.71 (0.38-1.03)	0.89 (0.34-1.44)	2.49 (1.91-3.07)	1.40 (0.94-1.86)	0.79 (0.27-1.31)	1.77 (1.33-2.22)
2013–15	0.92 (0.54-1.29)	1.61 (0.86-2.35)	3.12 (2.49-3.76)	1.49 (1.01-1.98)	1.15 (0.52-1.77)	2.44 (1.93-2.95)
2016–18	0.87 (0.51-1.24)	1.76 (0.97-2.55)	4.00 (3.30-4.70)	1.29 (0.84-1.73)	1.10 (0.48-1.72)	2.58 (2.07-3.10)
2019–21	0.75 (0.41-1.09)	0.95 (0.36-1.53)	2.59 (2.04-3.14)	2.04 (1.48-2.60)	1.57 (0.83-2.32)	2.36 (1.88-2.84)

CRC-All: all histologies, ADC: adenocarcinoma, NEN: neuroendocrine neoplasm

© 2025 Jooste V et al. *JAMA Network Open*

eTable 2: Model-based estimates of specific incidence rates (95% confidence interval) of colorectal cancer (/100,000) by histopathological type and tumor extension for the years 2004, 2013, and 2021 by sex

Histology / extension	Specific incidence (95% CI) Males			Specific incidence (95% CI) Females		
	2004	2013	2021	2004	2013	2021
**CRC-All**						
**15–39 yrs**	2.16 (1.83;2.49)	2.81 (2.54;3.08)	3.35 (2.89;3.81)	2.54 (2.17;2.91)	3.40 (3.10;3.70)	4.16 (3.61;4.70)
**40–49 yrs**	17.63 (16.01;19.25)	18.69 (17.61;19.78)	18.55 (16.88;20.23)	15.43 (13.98;16.88)	16.84 (15.82;17.86)	17.15 (15.58;18.72)
**≥50 yrs**	204.11 (197.83;210.40)	192.42 (188.91;195.92)	171.96 (166.95;176.96)	132.03 (127.72;136.33)	128.12 (125.69;130.55)	117.48 (113.88;121.08)
**ADC**						
**15–39 yrs**	1.92 (1.59;2.25)	2.11 (1.88;2.35)	2.2 (1.83;2.56)	1.94 (1.60;2.27)	2.18 (1.95;2.42)	2.32 (1.94;2.70)
**40–49 yrs**	16.96 (15.36;18.56)	17.37 (16.33;18.42)	16.94 (15.35;18.53)	14.90 (13.46;16.34)	15.67 (14.69;16.65)	15.65 (14.16;17.14)
**≥50 yrs**	201.82 (195.56;208.09)	188.48 (185.00;191.95)	169.28 (164.3;174.27)	130.47 (126.18;134.75)	125.09 (122.69;127.5)	115.02 (111.45;118.59)
ADC M0						
15–39 yrs	*1.36 (1.07;1.64)*	*1.41 (1.22;1.60)*	*1.36 (1.08;1.65)*	*1.32 (1.05;1.60)*	*1.41 (1.22;1.60)*	*1.4 (1.11;1.68)*
40–49 yrs	*12.48 (11.09;13.88)*	*12.14 (11.27;13.01)*	*11.13 (9.87;12.39)*	*10.8 (9.56;12.04)*	*10.77 (9.95;11.58)*	*10.09 (8.93;11.26)*
≥50 yrs	*147.6 (142.22;152.97)*	*136.37 (133.41;139.33)*	*119.49 (115.31;123.66)*	*95.36 (91.68;99.03)*	*90.33 (88.28;92.37)*	*80.92 (77.94;83.90)*
ADC M1						
15–39 yrs	*0.52 (0.36;0.68)*	*0.68 (0.55;0.81)*	*0.79 (0.56;1.01)*	*0.55 (0.38;0.72)*	*0.76 (0.61;0.90)*	*0.90 (0.65;1.16)*
40–49 yrs	*4.23 (3.47;4.99)*	*5.24 (4.66;5.82)*	*5.76 (4.79;6.73)*	*3.73 (3.04;4.41)*	*4.80 (4.25;5.35)*	*5.46 (4.53;6.39)*
≥50 yrs	*46.93 (43.96;49.91)*	*49.59 (47.80;51.37)*	*47.26 (44.60;49.91)*	*29.51 (27.52;31.49)*	*32.41 (31.19;33.63)*	*31.97 (30.07;33.87)*
**NEN**						
**15–39 yrs**	0.31 (0.18;0.44)	0.81 (0.63;0.99)	0.81 (0.55;1.06)	0.50 (0.30;0.70)	1.46 (1.20;1.73)	1.58 (1.17;20.00)
**40–49 yrs**	0.58 (0.29;0.87)	1.37 (1.02;1.71)	1.24 (0.76;1.71)	0.44 (0.21;0.67)	1.14 (0.83;1.45)	1.13 (0.69;1.56)
**≥50 yrs**	1.51 (1.05;1.98)	3.21 (2.78;3.65)	2.64 (2.09;3.18)	1.01 (0.69;1.34)	2.37 (2.04;2.70)	2.13 (1.68;2.57)
NEN M0						
15–39 yrs	*0.24 (0.12;0.36)*	*0.73 (0.55;0.91)*	*0.64 (0.41;0.86)*	*0.45 (0.25;0.66)*	*1.44 (1.16;1.73)*	*1.31 (0.92;1.70)*
40–49 yrs	*0.37 (0.14;0.60)*	*1.15 (0.81;1.48)*	*1.01 (0.57;1.45)*	*0.29 (0.1;0.48)*	*0.94 (0.64;1.24)*	*0.86 (0.47;1.25)*
≥50 yrs	*0.82 (0.49;1.14)*	*2.19 (1.83;2.55)*	*1.71 (1.28;2.14)*	*0.59 (0.34;0.83)*	*1.65 (1.37;1.93)*	*1.33 (0.99;1.68)*
NEN M1						
15–39 yrs	0.02 (0;0.05)	0.04 (0;0.07)	0.03 (0;0.07)	0.01 (0;0.03)	0.03 (0;0.06)	0.03 (0;0.06)
40–49 yrs	0.12 (0;0.26)	0.18 (0.07;0.29)	0.11 (0;0.23)	0.09 (0;0.19)	0.15 (0.05;0.24)	0.1 (0;0.21)
≥50 *yrs*	0.45 (0.19;0.72)	0.91 (0.67;1.14)	0.76 (0.44;1.07)	0.28 (0.11;0.46)	0.65 (0.47;0.83)	0.62 (0.36;0.88)

CRC-All: all histologies, ADC: adenocarcinoma, NEN: neuroendocrine neoplasm, M0: no distant metastasis, M1: distant metastasis

© 2025 Jooste V et al. *JAMA Network Open*

eTable 3: Model-based estimates of specific incidence rates (95% confidence interval) of colorectal adenocarcinoma (/100,000) by TNM stage and age class for the years 2004, 2013, and 2021 by sex

	Males			Females		
	**2004**	**2013**	**2021**	**2004**	**2013**	**2021**
**Stage I**						
**15–39 yrs**	0.33 (0.19,0.46)	0.34 (0.25,0.44)	0.34 (0.2,0.47)	0.36 (0.22,0.51)	0.42 (0.32,0.52)	0.45 (0.28,0.62)
**40–49 yrs**	3.90 (3.11,4.70)	3.41 (2.96,3.87)	2.88 (2.27,3.49)	3.41 (2.7,4.13)	3.31 (2.86,3.76)	3.05 (2.41,3.69)
**≥50 yrs**	45.71 (42.63,48.8)	41.97 (40.28,43.66)	36.94 (34.58,39.30)	21.77 (20.09,23.44)	22.13 (21.16,23.10)	21.33 (19.84,22.82)
**Stage II**						
**15–39 yrs**	0.49 (0.32,0.67)	0.50 (0.39,0.62)	0.46 (0.29,0.62)	0.43 (0.27,0.59)	0.44 (0.33,0.55)	0.41 (0.26,0.55)
**40–49 yrs**	3.99 (3.19,4.8)	3.93 (3.43,4.43)	3.48 (2.77,4.19)	3.16 (2.5,3.83)	3.15 (2.71,3.59)	2.82 (2.22,3.41)
**≥50 yrs**	52.04 (48.85,55.23)	47.55 (45.8,49.29)	39.43 (37.06,41.81)	35.84 (33.55,38.13)	33.11 (31.86,34.37)	27.74 (25.99,29.48)
**Stage III**						
**15–39 yrs**	0.50 (0.33,0.67)	0.51 (0.4,0.63)	0.51 (0.33,0.68)	0.52 (0.34,0.69)	0.53 (0.41,0.64)	0.52 (0.34,0.69)
**40–49 yrs**	4.41 (3.59,5.22)	4.50 (3.96,5.03)	4.41 (3.59,5.23)	4.04 (3.29,4.80)	4.09 (3.59,4.59)	3.98 (3.23,4.74)
**≥50 yrs**	42.56 (39.74,45.38)	37.32 (35.81,38.83)	32.01 (29.88,34.14)	31.67 (29.52,33.82)	27.54 (26.4,28.68)	23.44 (21.83,25.06)
**Stage IV (M1)**						
**15–39 yrs**	0.52 (0.36,0.68)	0.68 (0.55,0.81)	0.79 (0.56,1.01)	0.55 (0.38,0.72)	0.76 (0.61,0.90)	0.90 (0.65,1.16)
**40–49 yrs**	4.23 (3.47,4.99)	5.24 (4.66,5.82)	5.76 (4.79,6.73)	3.73 (3.04,4.41)	4.80 (4.25,5.35)	5.46 (4.53,6.39)
**≥50 yrs**	46.93 (43.96,49.91)	49.59 (47.8,51.37)	47.26 (44.6,49.91)	29.51 (27.52,31.49)	32.41 (31.19,33.63)	31.97 (30.07,33.87)

© 2025 Jooste V et al. *JAMA Network Open*

eTable 4: Model-based Annual Percent Changes (95% confidence interval) in colorectal adenocarcinoma by TNM stage for the years 2004, 2013 and 2021 by age class and sex

TNM Stage	APC, % (95%CI) in Males between:			APC, % (95%CI) in Females between:		
	2004 and 2013	2013 and 2021	2004 and 2021	2004 and 2013	2013 and 2021	2004 and 2021
**Stage I**						
15–39 yrs	0.5% (-3.5 to 4.6)	-0.2% (-3.7 to 3.5)	0.2% (-3.5 to 4.0)	1.6% (-2.4 to 5.8)	1.0% (-2.6 to 4.6)	1.3% (-2.4 to 5.2)
40–49 yrs	-1.5% (-3.6 to 0.7)	-2.1% (-4.0 to -0.1)	-1.8% (-3.7 to 0.2)	-0.4% (-2.5 to 1.9)	-1.0% (-2.9 to 1.0)	-0.7% (-2.6 to 1.3)
≥50 yrs	-0.9% (-1.9 to 0.1)	-1.6% (-2.4 to -0.7)	-1.2% (-1.9 to -0.6)	0.2% (-0.9 to 1.3)	-0.5% (-1.4 to 0.5)	-0.1% (-0.9 to 0.6)
**Stage II**						
15–39 yrs	0.2% (-3.4 to 3.9)	-1.2% (-4.4 to 2.2)	-0.5% (-3.8 to 3.0)	0.3% (-3.3 to 4.0)	-1.0% (-4.2 to 2.3)	-0.3% (-3.7 to 3.2)
40–49 yrs	-0.2% (-2.3 to 2.0)	-1.5% (-3.4 to 0.4)	-0.8% (-2.7 to 1.2)	-0.1% (-2.2 to 2.1)	-1.4% (-3.3 to 0.6)	-0.7% (-2.6 to 1.3)
≥50 yrs	-1.0% (-1.9 to -0.1)	-2.3% (-3.1 to -1.5)	-1.6% (-2.2 to -1.0)	-0.9% (-1.8 to 0.0)	-2.2% (-3.0 to -1.4)	-1.5% (-2.1 to -0.9)
**Stage III**						
15–39 yrs	0.3% (-3.1 to 3.8)	-0.2% (-3.2 to 3.0)	0.1% (-3.1 to 3.3)	0.2% (-3.2 to 3.7)	-0.3% (-3.3 to 2.9)	-0.0% (-3.2 to 3.2)
40–49 yrs	0.2% (-1.7 to 2.2)	-0.2% (-2.0 to 1.6)	0.0% (-1.8 to 1.8)	0.1% (-1.8 to 2.1)	-0.3% (-2.1 to 1.5)	-0.1% (-1.9 to 1.7)
≥50 yrs	-1.4% (-2.4 to -0.5)	-1.9% (-2.8 to -1.0)	-1.7% (-2.3 to -1.0)	-1.5% (-2.5 to -0.6)	-2.0% (-2.9 to -1.1)	-1.8% (-2.4 to -1.1)
**Stage IV (M1)**						
15–39 yrs	3.1% (-0.0 to 6.2)	1.8% (0.5 to 3.2)	2.5% (-0.4 to 5.4)	3.5% (0.4 to 6.7)	2.3% (1.0 to 3.6)	2.9% (0.1 to 5.9)
40–49 yrs	2.4% (0.5 to 4.4)	1.2% (0.3 to 2.1)	1.8% (0.1 to 3.6)	2.9% (0.9 to 4.8)	1.6% (0.7 to 2.5)	2.3% (0.6 to 4.0)
≥50 yrs	0.6% (-0.3 to 1.5)	-0.6% (-1.0 to -0.2)	0.0% (-0.6 to 0.6)	1.0% (0.1 to 2.0)	-0.2% (-0.6 to 0.2)	0.5% (-0.2 to 1.1)

© 2025 Jooste V et al. *JAMA Network Open*

eTable 5: Annual percent change (95% confidence interval) in colorectal cancer by age class, histopathological type and tumor extension (males and females pooled). Model-based estimates from flexible model and Joinpoint regression

Histology / extension	APC, % (95%CI)											
	Flexible model	Joinpoint	Flexible model		Joinpoint APC by segment							
	Overall APC	AAPC	APC		Segment 1		Segment 2		Segment 3		Segment 4	
	2004-21	2004-21	2004-13	2013-21	endpoints	APC	endpoints	APC	endpoints	APC	endpoints	APC
**CRC-All**												
**15-39 yrs**	2.8 (1.4 to 4.2)	3.0 (0.9 to 5.2)	3.2 (1.7 to 4.7)	2.4 (1.1 to 3.7)	nj							
**40-49 yrs**	0.5 (-0.4 to 1.3)	0.8 (0.0 to 1.7)	0.8 (-0.2 to 1.8)	0.1 (-0.8 to 0.9)	nj							
**≥50 yrs**	-0.8 (-1.1 to -0.6)	-0.5 (-0.9 to -0.1)	-0.5 (-0.9 to -0.1)	-1.2 (-1.6 to -0.9)	nj							
**ADC**												
**15-39 yrs**	0.9 (-0.7 to 2.5)	1.3 (-1.6 to 4.3)	1.2 (-0.5 to 2.9)	0.6 (-0.9 to 2.2)	nj							
**40-49 yrs**	0.1 (-0.8 to 1.0)	0.5 (-0.3 to 1.3)	0.4 (-0.6 to 1.4)	-0.2 (-1.1 to 0.7)	nj							
**≥50 yrs**	-0.9 (-1.1 to -0.6)	-0.6 (-0.9 to -0.2)	-0.6 (-1.0 to -0.2)	-1.2 (-1.6 to -0.8)	nj							
**ADC M0**												
**15-39 yrs**	0.2 (-1.8 to 2.1)	0.5 (-2.9 to 4.2)	0.5 (-1.5 to 2.7)	-0.2 (-2.1 to 1.6)	nj							
**40-49 yrs**	-0.5 (-1.6 to 0.5)	0 (-1 to 1)	-0.2 (-1.3 to 1.0)	-0.9 (-2.0 to 0.1)	nj							
**≥50 yrs**	-1.1 (-1.4 to -0.8)	-0.6 (-1.1 to -0.2)	-0.7 (-1.2 to -0.2)	-1.5 (-1.9 to -1.1)	nj							
**ADC M1**												
**15-39 yrs**	2.7 (-0.1 to 5.6)	2.5 (-0.7 to 6.2)	3.3 (0.2 to 6.5)	2.1 (-0.6 to 4.8)	nj							
**40-49 yrs**	2.0 (0.4 to 3.7)	2.0 (0.3 to 3.8)	2.6 (0.7 to 4.6)	1.4 (-0.3 to 3.1)	nj							
**≥50 yrs**	0.3 (-0.3 to 0.8)	0.3 (-0.4 to 1.0)	0.8 (-0.1 to 1.7)	-0.4 (-1.1 to 0.3)	2004-2009	1.8 (0 to 8.9)	2009-2021	-0.3 (-5.6 to 0.5)				
**NEN**												
**15-39 yrs**	6.6 (3.1 to 10.2)	5.6 (-10.0 to 23.9)	12.1 (6.9 to 17.6)	0.6 (-2.6 to 4.0)	2004-2008	-11.2 (-45.7 to 45.2)	2008-2012	44.1 (-18.7 to 155.6)	2012-2021	-0.6 (-7.5 to 6.7)		
**40-49 yrs**	5.1 (0.6 to 9.8)	3.6 (-11.5 to 21.2)	10.6 (4.6 to 16.9)	-0.7 (-4.9 to 3.6)	2004-2007	-21.9 (-64.3 to 71)	2007-2013	23.9 (-6.9 to 64.8)	2013-2021	0.8 (-9.7 to 12.4)		
**≥50 yrs**	3.9 (1.3 to 6.5)	5.0 (3.0 to 7.4)	9.3 (4.9 to 13.9)	-1.9 (-4.3 to 0.6)	2004-2016	11.1 (8.6 to 15.6)	2016-2021	-8.3 (-19 to -2.1)				
**NEN M0**												
**15-39 yrs**	6.3 (2.2 to 10.4)	6.2 (1.3 to 12.4)	13.5 (7.2 to 20.3)	-1.4 (-5.0 to 2.4)	2004-2009	-6.5 (-48.3 to 25.3)	2009-2012	73.6 (-11.3 to 128.5)	2012-2021	-3.2 (-11.7 to 7.6)		
**40-49 yrs**	6.3 (0.8 to 12.1)	2.9 (-0.6 to 8.9)	13.6 (6.0 to 21.7)	-1.3 (-6.2 to 3.8)	2004-2007	-41.3 (-71.8 to -8.1)	2007-2013	46.2 (27.9 to 149.9)	2013-2021	-2.3 (-11.6 to 4.4)		
**≥50 yrs**	4.7 (1.4 to 8.1)	3.7 (-5.9 to 14.3)	11.9 (6.1 to 17.9)	-2.8 (-5.7 to 0.2)	2004-2007	-4.7 (-38 to 46.4)	2007-2013	22.3 (4.5 to 43.1)	2013-2019	2.1 (-7.4 to 12.6)	2019-2021	-24.8 (-55.5 to 27.3)
**NEN M1**												
**15-39 yrs**	3.3 (-10.9 to 19.7)	nc	8.2 (-8.4 to 27.7)	-2 (-15.0 to 13.0)	nc							
**40-49 yrs**	0.1 (-9.7 to 11.0)	nc	4.9 (-7.2 to 18.6)	-5 (-14.3 to 5.4)	nc							

© 2025 Jooste V et al. *JAMA Network Open*

**≥50 yrs**	3.8 (-0.9 to 8.8)	nc	8.7 (0.4 to 17.7)	-1.5 (-6.4 to 3.7)	nc

Abbreviation: CRC-All: all histologies, ADC: adenocarcinoma, NEN: neuroendocrine neoplasm, M0: no distant metastasis, M1: distant metastasis

© 2025 Jooste V et al. *JAMA Network Open*

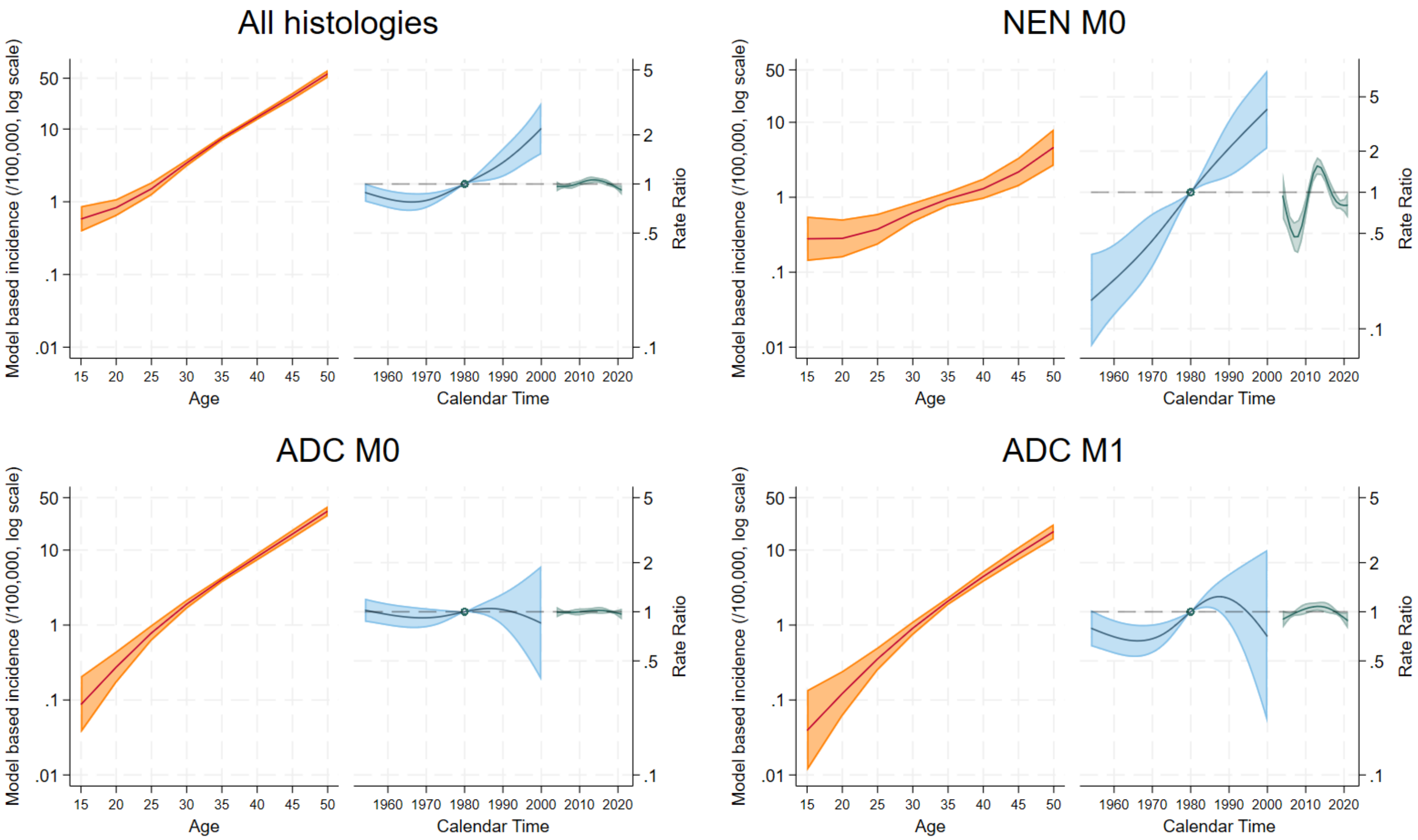

eFigure: Effects of age, period and cohort, by histopathological type and tumor extension (age 15–49)

Age-period-cohort flexible models with drift term included in the cohort effect, colorectal cancer incidence in log scale given /100,000 inhabitants between 2004 and 2021. For each panel, the leftmost orange line refers to the estimated age effect, the middle blue line to the estimated cohort effect, and the rightmost green line to the estimated period effect. The respective regions surrounding the lines provide the 95% confidence intervals. The circle indicates the reference cohort (birth in 1980).
ADC: adenocarcinoma, NEN: neuroendocrine neoplasm, M0: non metastatic, M1: distant metastasis

© 2025 Jooste V et al. *JAMA Network Open*
