# Supplementary material for: Epidemiological Classification Changes and Incidence of Early-Onset Colorectal Cancer
Source: JAMA Netw Open. 2025 Nov 5;8(11):e2541732. doi: 10.1001/jamanetworkopen.2025.41732 (PMC12590295; doi:10.1001/jamanetworkopen.2025.41732)
Supplement: Supplement 2. — Nonauthor Collaborators [file jamanetwopen-e2541732-s002.pdf]

Supplemental Online Content: Nonauthor Collaborators
*First name, last name, and suffix (if applicable) are required and will appear in PubMed.

***Group Name(s): French Network of Cancer Registries (FRANCIM)**

***First Name and Middle Initial(s)**	***Last Name**	***Suffix (eg, Jr, III)**	Academic Degrees	Institution	Location (city, state/province, country)	Role or Contribution, eg, chair, principal investigator	Group (if more than 1 Group listed in the byline) and/or Subgroup (eg, Steering Committee)
Arnaud	Alves		MD PhD	Registre des cancers digestifs du Calv	Caen	member	FRANCIM
Isabelle	Baldi		MD PhD	Registre des tumeurs primitives du Système Nerveux Central	Bordeaux	member	FRANCIM
Simona	Bara		MD	Registre des cancers de la Manche	Cherbourg	member	FRANCIM
Anne-Marie	Bouvier		MD PhD	Registre Bourguignon des cancers dig	Dijon	member	FRANCIM
Véronique	Bouvier		MD	Registre des cancers digestifs du Calv	Caen	member	FRANCIM
Emmanuel	Chirpaz		MD PhD	Registre des cancers de la Réunion	Saint Denis	member	FRANCIM
Jacqueline	Clavel		MD PhD	Registre National des Hémopathies de l'Enfant	Paris	member	FRANCIM
Gaëlle	Coureau		MD PhD	Registre général des cancers de la Gironde	Bordeaux	member	FRANCIM
Anne	Cowppli-Bony		MD PhD	Registre des tumeurs de Loire-Atlantique/Vendée	Nantes	member	FRANCIM
Sandrine	Dabakuyo-Yonli		MD PhD	Registre des cancers du sein et des ca	Dijon	member	FRANCIM
Laetitia	Daubisse-Marliac		MD	Registre des cancers généraux du Tar	Albi	member	FRANCIM
Patricia	Delafosse		MD	Registre général des cancers de l'Isèr	Grenoble	member	FRANCIM
Emmanuel	Desandes		MD PhD	Registre National des Tumeurs solides de l'Enfant	Nancy	member	FRANCIM
Jacqueline	Deloumeaux		MD PhD	Registre des Cancers de la Guadeloupe	Pointe-à-Pitre	member	FRANCIM
Kinan	Drak Alsibai		MD PhD	Registre des cancers de Guyane	Cayenne	member	FRANCIM
Pascale	Grosclaude		MD PhD	Registre des cancers généraux du Tar	Albi	member	FRANCIM
Adrien	Guilloteau		MD PhD	Registre des Hémopathies Malignes de Côte d'Or	Dijon	member	FRANCIM

Supplemental Online Content: Nonauthor Collaborators
*First name, last name, and suffix (if applicable) are required and will appear in PubMed.

*First Name and Middle Initial(s)	*Last Name	*Suffix (eg, Jr, III)	Academic Degrees	Institution	Location (city, state/province, country)	Role or Contribution, eg, chair, principal investigator	Group (if more than 1 Group listed in the byline) and/or Subgroup (eg, Steering Committee)
Karima	Hammas		MD PhD	Registre des cancers du Haut-Rhin	Mulhouse	member	FRANCIM
Florent	Huré		MD PhD	Registre général des cancers de Haut	Limoges	member	FRANCIM
Clarisse	Joachim		MD PhD	Registre des Cancers de la Martinique	Fort de France	member	FRANCIM
Valérie	Jooste		PhD	Registre Bourguignon des cancers dig	Dijon	member	FRANCIM
Brigitte	Lacour		MD PhD	Registre National des Tumeurs solides de l'Enfant	Nancy	member	FRANCIM
Bénédicte	Lapôtre-Ledoux		MD	(Registre général des cancers de la Somme		member	FRANCIM
Florence	Molinié		MD PhD	Registre des tumeurs de Loire-Atlantique/Vendée	Nantes	member	FRANCIM
Marc	Maynadié		MD PhD	Registre des Hémopathies Malignes de Côte d'Or	Dijon	member	FRANCIM
Alain	Monnereau		MD PhD	Registre des Hémopathies Malignes de Gironde	Bordeaux	member	FRANCIM
Jean-Baptiste	Nousbaum		MD PhD	Registre Finistérien des tumeurs dige	Brest	member	FRANCIM
Sandrine	Plouvier		MD PhD	Registre des cancers de Lille et de sa	Lille	member	FRANCIM
Claire	Poulalhon		MD PhD	Registre National des Hémopathies de l'Enfant	Paris	member	FRANCIM
Arnaud	Seigneurin		MD PhD	Registre général des cancers de l'Isère	Grenoble	member	FRANCIM
Xavier	Troussard			Registre des Hémopathies malignes de Basse Normandie	Caen	member	FRANCIM
Thomas	Systchenko		MD PhD	Registre des cancers de Poitou–Charentes	Poitiers	member	FRANCIM
Brigitte	Trétarre		MD	Registre général des tumeurs de l'Hérault	Montpellier	member	FRANCIM
Michel	Velten		MD PhD	Registre des Cancers du Bas Rhin	Strasbourg	member	FRANCIM
Nicolas	Vigneron		MD PhD	Registre Général des cancers du Calva	Caen	member	FRANCIM

Supplemental Online Content: Nonauthor Collaborators
*First name, last name, and suffix (if applicable) are required and will appear in PubMed.

***First Name and Middle Initial(s)**	***Last Name**	***Suffix (eg, Jr, III)**	Academic Degrees	Institution	Location (city, state/province, country)	Role or Contribution, eg, chair, principal investigator	Group (if more than 1 Group listed in the byline) and/or Subgroup (eg, Steering Committee)
Anne-Sophie	Woronoff		MD PhD	Registre des tumeurs du Doubs	Besancon	member	FRANCIM
