# Supplementary material for: Epidemiological Classification Changes and Incidence of Early-Onset Colorectal Cancer
Source: JAMA Netw Open. 2025 Nov 5;8(11):e2541732. doi: 10.1001/jamanetworkopen.2025.41732 (PMC12590295; doi:10.1001/jamanetworkopen.2025.41732)
Supplement: Supplement 3. — Data Sharing Statement [file jamanetwopen-e2541732-s003.pdf]

Data Sharing Statement

Jooste. Epidemiological Classification Changes and Incidence Increases in Early-Onset Colorectal Cancer. *JAMA Netw Open*. Published November 05, 2025. doi:10.1001/jamanetworkopen.2025.41732

Data
Data available: No

Additional Information
Explanation for why data not available: Data accessibility: Anonymized data that are minimally required to replicate the outcomes of the study can be obtained on request to the corresponding author.
